# Interleukin-1 beta: a potential link between stress and the development of visceral obesity

**DOI:** 10.1186/1472-6793-12-8

**Published:** 2012-06-27

**Authors:** Kristin J Speaker, Monika Fleshner

**Affiliations:** 1 Department of Integrative Physiology, University of Colorado at Boulder, 1725 Pleasant Street, Boulder Colorado, 80309, USA

## Abstract

**Background:**

A disproportionate amount of body fat within the abdominal cavity, otherwise known as visceral obesity, best predicts the negative health outcomes associated with high levels body fat. Growing evidence suggests that repeated activation of the stress response can favor visceral fat deposition and that visceral obesity may induce low-grade, systemic inflammation which is etiologically linked to the pathogenesis of obesity related diseases such as cardiovascular disease and type 2 diabetes. While the obesity epidemic has fueled considerable interest in these obesity-related inflammatory diseases, surprisingly little research is currently focused on understanding the functions of inflammatory proteins in healthy, non-obese white adipose tissue (WAT) and their possible role in modulating stress-induced shifts in body fat distribution.

**Hypothesis:**

The current review presents evidence in support the novel hypothesis that stress-evoked interleukin-1 beta (IL-1β) signaling within subcutaneous adipose tissue, when repeatedly induced, contributes toward the development of visceral obesity. It is suggested that because acute stressor exposure differentially increases IL-1β levels within subcutaneous adipose relative to visceral adipose tissue in otherwise healthy, non-obese rats, repeated induction of this response may impair the ability of subcutaneous adipose tissue to uptake energy substrates, synthesize and retain triglycerides, and/or adapt to positive energy balance via hyperplasia. Consequently, circulating energy substrates may be disproportionately shunted to visceral adipose tissue for storage, thus driving the development of visceral obesity.

**Conclusions:**

This review establishes the following key points: 1) body fat distribution outweighs the importance of total body fat when predicting obesity-related disease risk; 2) repeated exposure to stress can drive the development of visceral obesity independent of changes in body weight; 3) because of the heterogeneity of WAT composition and function, an accurate understanding of WAT responses requires sampling multiple WAT depots; 4) acute, non-pathogenic stressor exposure increases WAT IL-1β concentrations in a depot specific manner suggesting an adaptive, metabolic role for this cytokine; however, when repeated, stress-induced IL-1β in non-visceral WAT may result in functional impairments that drive the development of stress-induced visceral obesity.

## Background

Advances in our understanding of the etiology of weight gain and the regulation of energy homeostasis have greatly contributed toward the widespread efforts to combat obesity. While it has become increasingly apparent that disproportionate amounts of visceral white adipose tissue (WAT) and a low-grade inflammatory state contribute to the pathogenesis of obesity [1-5] the mechanisms that regulate the distribution of body fat and the functions of inflammatory proteins in healthy, non-obese WAT remain unclear. Interestingly, stressor exposure and immunity have both been found to impact metabolism and to be linked to the development of visceral obesity [5-13]. Despite this evidence, little attention has been paid to the metabolic effects of cytokines in healthy, non-obese adipose tissue or the potential effects of repeated stress on WAT function. Herein we present new data that inflammatory proteins are elevated by non-pathogenic stress resulting in depot-specific shifts in the local cytokine milieu of non-obese WAT [14] supporting the hypothesis that the immune system may play a role in the regulation of body fat distribution through depot specific immune-metabolic interactions. This review presents evidence that repeated exposure to stress contributes to the development of visceral obesity and that stress-induced cytokine production may play an integral role in this maladaptive effect. We begin by arguing that body fat distribution outweighs the importance of total body fat followed by a discussion of stress-induced visceral obesity. We then present data in support of the hypothesis that acute, stress-induced shifts in non-visceral WAT cytokines serve adaptive functions that become maladaptive when repeated. More specifically, we hypothesize that stress-evoked elevations impair the ability of non-visceral WAT to uptake, resynthesize and retain lipids, and/or to expand in the face of positive energy balance and that these cytokine-driven impairments consequently contribute to the development of visceral obesity.

### Body fat distribution and obesity-related disease risk

Obesity, or excess WAT, affects more than 33 % of the American population today [15] but is excess body fat really bad for you? Although studies demonstrate that chronic non-communicable diseases such as type 2 diabetes and cardiovascular disease coincide with the rise in obesity not all forms of obesity are associated with metabolic syndrome and chronic-disease development [16-19]. What’s more, current epidemiological research suggests that the way in which WAT is distributed throughout the body is a better predictor of obesity-related disease risk than total body fat mass [1-4]. Numerous studies have demonstrated that obesity-related health risk in humans is strongly correlated with an increased ratio of visceral fat mass relative to non-visceral fat mass (i.e. increased anthropometric measures such as waist-circumference and waist-to- hip ratios) suggesting that this is a maladaptive body fat distribution reflective of visceral obesity [20-22]. A worldwide case–control study done by Yusuf et al. (2005), for example, demonstrated that the waist-to-hip circumference ratio - an indirect estimate of visceral obesity - far exceeds Body Mass Index (BMI = body weight (kg)/height (m)^2^) in its association with myocardial infarction risk [1]. Evidence also conversely demonstrates that a reduced ratio of visceral fat mass relative to non-visceral fat mass is correlated with reduced disease risk [23,24]. Furthermore, while a disproportionate accumulation of visceral WAT is correlated with chronic low-grade inflammation and pathogenesis, the opposite appears to be true for disproportionate amounts of non-visceral WAT [3,25-27]. Manolopoulos et al. (2010) recently reviewed the protective properties of gluteofemoral fat in humans (i.e. WAT stored in the thighs and hips) suggesting that these non-visceral, subcutaneous fat depots act as a ‘metabolic sink’ for the daily influx and long term storage of dietary lipids [26]. In other words, to the degree it can effectively uptake circulating energy substrates such as lipids and glucose, resynthesize and retain triglycerides (the storage form of energy in WAT) and expand in response to positive energy balance, non-visceral WAT - or subcutaneous WAT in humans - is thought to protect against the development of visceral obesity. Collectively these studies corroborate epidemiological evidence further demonstrating that body fat distribution outweighs the importance of total body fat when predicting obesity-related disease risk. What factors, then, lead to the disproportionate accumulation of visceral body fat?

### Visceral obesity: the stress hypothesis

As with any long-term physiological response, the development of visceral obesity occurs as a result of complex interactions between genetic and environmental factors [28]. A summary of factors known to affect body fat distribution is presented in Table 1.

**Table 1:** Factors associated with the development of visceral obesity

*Factor*	*Association*	*Reference*
Genetics	Ethnicity, hypothalamic genetic disorders, etc.	[28-32]
Age	↑ Age → ↑ WC (Age > 60 → ↑ WC)	[33,34]
Sex	Male WC > Female WC	[33]
Physical Activity Status	↑ Physical Activity Status → ↓ WC	[35,36]
Dietary Composition	↑ Fat/Fructose content → ↑ WHR	[37-39]
Smoking	↑ Smoking → ↑ WHR	[8,40]
Alcohol Consumption	↑ Alcohol consumption → ↑ WHR	[8,41]
Socio-Economic Status	↓ Socio-Economic Status → ↑ WHR	[8,42]
Stress-related Mood	↑ Depression/Anxiety → ↑ WHR	[8,43]
Disorders		

Abbreviations: *WC*, waist-circumference; *WHR*, waist-to-hip circumference ratio.

Notably, life stress, or the real or imagined experience of an adverse event [44] negatively impacts nearly all of the factors associated with the development of visceral obesity (Table 1). For example, high levels of life stress are linked to poor dietary habits [45], addictive behaviors such as smoking and alcohol consumption [46-48], a low socio-economic status [8], and an increased prevalence of mood disorders such as depression and anxiety [49].

The notion that visceral obesity may be a physiological adaptation to chronic or repeated stress first came to fruition in the early 1980s through the clinical research of Per Björntorp [50,51]. Along with collaborator Roland Rosmond, Björntorp observed that individuals with low economic status who were repeatedly subjected to psychosocial and economic stressors developed both perturbations in hypothalamic-pituitary-adrenal (HPA) axis function and increased visceral adiposity [42,50]. Björntorp and Rosmond accordingly hypothesized that disproportionate gains in visceral fat mass may be due to an increase in HPA axis activity induced by repeated exposure to stressors. Their subsequent work laid the foundation for stress-induced visceral obesity by demonstrating that: (a) the HPA axis is finely tuned to and in connection with an individual’s real or perceived environment [9,52]; (b) frequent, repeated activation of the HPA axis often leads to dysregulated HPA activity as evidence by low diurnal variability with elevated and/or sustained glucocorticoid responses to an acute stressor and/or a dampened negative feedback response stress following an injection of dexamethasone (a glucocorticoid agonist) [9,53,54]; and (c) dysregulation of the HPA axis is positively correlated with visceral obesity [9].

Additional support for the stress hypothesis can be found in epidemiological studies demonstrating a correlation between waist-to-hip circumference ratio, low socio-economic status (i.e. low income and/or low education level) [15,55] and job stress [42,56]. Elevated waist-to-hip circumference ratios are also associated with stress-related mood disorders such as anxiety and depression [57]. Potential causative factors in the observed association between stress and visceral obesity include increased glucocorticoid activity in WAT depots [58], excessive vulnerability to the external environment due to the sustained and uncontrollable stress of poverty and/or threatening social pressures [59-61], and - due to their low cost and high palatability - increased consumption of foods that are high in fat and/or glycemic load and of a low nutritional value [45,60,61]. In spite of these correlative observations the pathogenic mechanisms linking stress with a central redistribution of body fat remain unclear. This is likely due to the fact that accurate assessment of visceral fat mass in humans can only be done through scan-based systems such as computed tomography and magnetic resonance imaging. Because they allow for the precise quantification of visceral fat mass through dissection, however, animal models serve as effective tools for investigating stress-induced visceral obesity [62,63].

Animal models of chronic stress include repeated exposure to physical restraint [64], conditioned fear [65], foot or tail-shock [66], novel or loud noises [64], social stress [67,68], or a combination of multiple stressors. In support of the stress hypothesis, animals exposed to chronic social stress often display maladaptive changes in their body fat distribution though this is rarely detected in the face of body weight gain [63,64]. Contrary to the high degree of body weight variability reported in chronically stressed humans [69] animals normally reduce their total body weight in response to repeated stressor exposure because they eat less in combination with the metabolic demands of the stress response [62,63,67]. Although these data superficially contradict the stress hypothesis, upon proper quantification of body fat distribution, chronically stressed animals often demonstrate a maladaptive shift in the distribution of their body fat stores irrespective of changes in body weight [67,70-74]. The development of visceral obesity may therefore not always be detected in animals exposed to repeated stress because a) visceral versus non-visceral fat depots remain unclearly defined; b) body fat distribution is assessed inconsistently or improperly and/or c) the lack of standardization with regard to the palatability of the diet. These inconsistent results highlight the importance of properly assessing and defining body fat distribution - not just total adiposity or individual fat pad weights - when evaluating the effects of stressor exposure on the development of visceral obesity.

### Clarifying the assessment of body fat distribution: visceral versus non-visceral WAT

Although it is widely accepted that body composition is defined as the mass of total body fat relative to total body weight, the definition of body fat distribution remains unclear in the literature. Most agree that it is quantified as the ratio of visceral fat mass relative to non-visceral fat mass or total body fat mass [29]; the ambiguities lay within the conflicting opinions about the depots that constitute visceral WAT [58].

Based upon their anatomical location, WAT depots are heterogeneous in their innervation pattern [75-77], composition [78-81] and function [81-83]. The predominant storage locations of WAT in mammals are the subcutaneous depots located between the epidermis and muscle and the intra-abdominal depots found within the peritoneal cavity [29]. For the intra-abdominal depots - which include the omental, mesenteric, gonadal, retroperitoneal and perirenal depots - further distinctions are made in reference to the circulatory system into which they drain [84]. Explicitly, the omental and mesenteric depots drain directly into the portal venous system while the gonadal, retroperitoneal, and perirenal depots drain into the general venous circulation [84,85]. Because of this quintessential distinction the omental and mesenteric depots are considered to be true visceral WAT; the subcutaneous depots and remaining intra-abdominal depots (gonadal, retroperitoneal, perirenal) thus constitute non-visceral WAT [84-86]. When visceral WAT mass is clearly defined as portal draining WAT, body fat distribution emerges as a quantifiable ratio of visceral WAT mass, or the weight of the omental and mesenteric depots, relative to non-visceral WAT mass (total body fat mass - visceral mass). By clearly defining visceral vs. non-visceral WAT, shifts in body fat distribution can now be quantified as changes in the ratio between visceral and non-visceral fat mass. Using this definition, visceral obesity becomes the point at which the ratio of visceral to non-visceral WAT becomes pathogenic, or associated with disease, and the development of visceral obesity the process whereby visceral WAT depots disproportionately expand relative to non-visceral WAT.

Despite the emergent importance of accurately assessing body fat distribution and understanding the etiology of visceral obesity, the bulk of obesity-related research remains focused on the causes and consequences of excessive body fat mass irrespective of its distribution. For example, it is clear that positive energy balance (energy input > energy expenditure) results in body fat gain and that factors affecting the energy balance equation contribute to the development or prevention of obesity [45,87,88]. It is also widely accepted that a state of low-grade systemic inflammation accompanies obesity- related diseases though whether low-grade systemic inflammation is a cause or consequence of obesity-related diseases remains unclear and highly debated [89]. In spite of the wealth of knowledge regarding the causes and consequences of general obesity, however, very little is currently understood about the mechanisms controlling the regional storage of excess energy or the functions cytokines serve in healthy, non-obese WAT. The subsequent sections of this review therefore present evidence in support of the novel hypothesis that the development of visceral obesity may be due to impairments in subcutaneous/non-visceral adipose function and that repeated stressor exposure may exert these effects through local, depot specific induction of cytokines.

## A novel mechanistic hypothesis for the development of visceral obesity

A maladaptive shift in body fat distribution does not require fat mass gain; it can occur in the face of weight stability or even weight loss. In other words, the development of visceral obesity can occur in one of three ways: 1) visceral fat mass expands to a greater relative extent than non-visceral fat mass, 2) non-visceral fat mass atrophies to a greater relative extent than visceral fat mass, or 3) a combination of the above. Changes in body fat distribution are consequently independent of total body fat mass and regulated by distinct mechanisms [50].

Ironically, despite the fact that body fat distribution is affected by both gains and/or losses in visceral and non-visceral adiposity, mechanistic research has focused on the pathways through which stress-induced perturbations in steroid hormone production impact visceral fat mass [6-12]. For that reason, little light has been shed upon the potential mechanisms whereby repeated stressor exposure impacts non-visceral fat mass and function. Considering non-visceral WAT protects against visceral obesity relative to its effectiveness as a storage depot [26], it is essential to consider the possibility that stress-induced visceral obesity occurs through a combination of maladaptive visceral and non-visceral effects.

### Visceral obesity is marked by impaired subcutaneous adipose tissue function

In healthy mammals, non-visceral WAT depots comprise the majority of total body fat. Consequently, impairments in non-visceral WAT function can significantly impact lipid deposition in ectopic or visceral depots. In other words, to properly act as a metabolic ‘sink’ or buffer against excess energy storage in alternative locations, non-visceral adipose tissue must be able to effectively uptake circulating energy substrates, (re)synthesize, store and retain triglyceride molecules, and expand in response to positive energy balance [26]. If any of these buffering functions (uptake, synthesis, storage, retention, expansion) become impaired, the effectiveness of the tissue to buffer excess energy is subsequently compromised. The potential for deposition in the visceral depots thus increases as energy substrates are shunted to alternative storage locations. In fact, data demonstrate that the capacity of subcutaneous adipose tissue to uptake energy and grow is inversely associated with visceral obesity [90].

**Figure 1:**
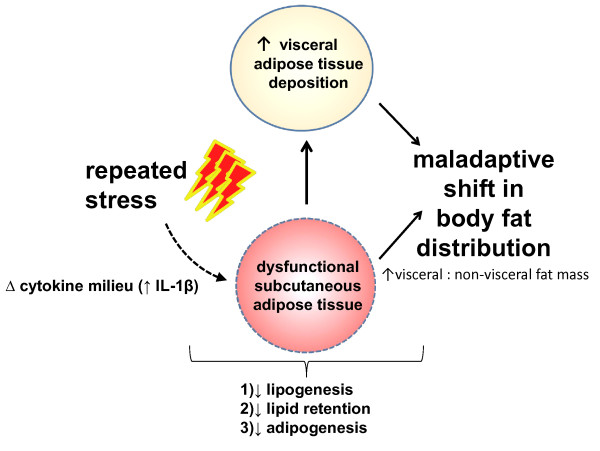
**Stress-induced impairments in subcutaneous WAT function contribute to the development of visceral obesity.** Exposure to acute stress alters local WAT IL-1β content in a depot specific manner. This depot specific response, when repeated, may lead to a maladaptive shift in the milieu of inflammatory proteins found within subcutaneous adipose tissue such that its ability to properly function becomes impaired. Consequently, circulating lipids are shunted towards to visceral adipose resulting in the development of visceral obesity as marked by a maladaptive shift in body fat distribution or an increase in the ratio of visceral to non-visceral fat mass.

In healthy non-obese humans, Rebuffe-Scrive et al. (1988) demonstrated that both fat cell size and lipoprotein lipase (LPL) activity (the enzyme which regulates the uptake of lipids from the bloodstream) were higher in femoral, non-visceral adipocytes than in abdominal adipocytes [90]. They further established that the difference in LPL activity between the abdominal and femoral depots was lost with the development of visceral obesity, but not with the development of non-visceral, lower body obesity [90]. Explicitly, a maladaptive shift in body fat distribution was marked by an increase in abdominal adipocyte LPL activity and a decrease in LPL activity in subcutaneous, femoral adipocytes [90]. Furthermore, Stanhope et al. (2011) recently demonstrated that a fructose laden diet increased visceral adipose deposition due to a dampened subcutaneous LPL response to fructose based meals [91]. The authors hypothesized these observations were due to the fact that subcutaneous LPL activation is significantly more sensitive to insulin than visceral LPL [92] and fructose produces a dampened insulin response relative to glucose [91]. Thus, considering the lipogenic function of LPL, these data strongly suggest that the development of visceral obesity is directly due to a reduction in the ability of subcutaneous adipose tissue to uptake circulating lipids as determined by LPL activity and local adipocyte insulin sensitivity. Healthy, non-obese organisms have visceral adipose tissue that expands predominantly via adipocyte hypertrophy - the mature adipocytes get larger - whereas their non-visceral and/or subcutaneous adipose tissue expands predominantly via hyperplasia, or the process whereby new, mature adipocytes are formed [82,93,94]. In contrast, viscerally obese organisms have subcutaneous depots that display dampened hyperplasia potential. A series of studies by Peinado et al. (2010) and Miranda et al. (2008) demonstrated that in lean individuals, lamin A and lamin C - essential proteins for preadipocyte differentiation - are typically over-expressed in the stroma-vascular cell fraction (i.e. non-adipocyte) of subcutaneous adipose tissue relative to visceral adipose tissue and this over-expression is lost when an individual becomes viscerally obese [95,96]. These data are highlighted because evidence suggests when a mature adipocyte reaches a certain size - as determined by its location - it signals the production of new adipocytes [97]. This process, termed adipogenesis, is tightly regulated by numerous factors and requires the commitment, proliferation and differentiation of resident preadipocytes [98]. If adipogenesis is impaired within subcutaneous adipose tissue its ability to adapt in the face of sustained, positive energy balance diminishes. Collectively these studies link visceral obesity to dysregulated subcutaneous WAT function suggesting this depot may play a pivotal role in the development of visceral obesity (Figure 1). We finish this review with evidence supporting the hypothesis that repeated stress-induced shifts in the cytokine milieu of non-visceral WAT impair subcutaneous function which contributes to the development of visceral obesity.

## Stress induced IL-1β: a potential link between stress and the development of visceral obesity

Adipose tissue is comprised of a multitude of cells such as preadipocytes, mature adipocytes, mast cells, endothelial cells, fibroblasts and numerous types of immune cells [80,99]. Recent findings suggest that the cross-talk between resident immune cells and adipocytes modulates adipocyte metabolism, preadipocyte differentiation [100,101] and innate immune function [13]. The link between immunity and adipose metabolism was pioneered in the late 1980s by Besedovsky and colleagues who demonstrated that cytokines such as IL-1β were capable of inducing endocrine and metabolic changes within the body [102]. More recent work demonstrates that both preadipocytes and mature adipocytes express innate immune receptors and respond to endotoxin stimulation with the production of cytokines, chemokines and adipokines [103]. Resident adipose tissue immune cells have also been shown to act as local metabolic regulators through the release of factors that alter adipocyte metabolism and differentiation [100,104,105]. Moreover, changes in the number and proportion of circulating lymphocytes [106,107] coupled with a rise in systemic inflammatory markers (i.e. cytokines and acute phase proteins [66,106,107]) illustrates that the immune system actively responds to acute stressor exposure [108]. Collectively these studies demonstrate that the immune system and adipose metabolism are tightly linked, each contributing to the function of the other and that activation of the stress response serves as a pathway whereby immune-metabolic cross-talk is initiated. What’s more, we have recent data demonstrating that inflammatory proteins in WAT are directly affected by stressor exposure in a depot specific manner.

### Acute stressor exposure affects WAT cytokine concentrations

**Figure 2:**
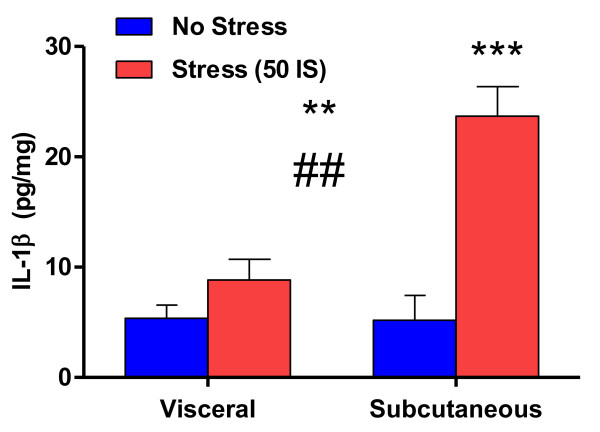
**IL-1β response to acute stressor exposure in visceral and subcutaneous WAT.** Effect of acute stressor exposure on the IL-1β protein content of visceral (omental) and subcutaneous (inguinal) WAT in healthy, non-obese rats. 21 week old male F344 rats were exposed to 50 inescapable 1.5 mA tail shocks (stress, n = 24) or remained in their home cages (no stress, n = 26). Rats were sacrificed immediately following stressor exposure. Approximately 0.3 g of omental and subcutaneous WAT depots were harvested, spot frozen in liquid nitrogen and processed via homogenization in a RIPA lysis buffer. IL-1β protein analysis (pg IL-1β per mg total WAT protein) was done via ELISA (R&D Systems Minneapolis, MN). Effect of depot on IL-1β concentration F(1,46) = 15.751, **P < 0.001. Effect of stress on IL-1β content: F(1,46) = 28.461, ***P < 0.0001. Depot by stress interaction: F(3,44) = 13.312, ##P < 0.001. Values represent group means + standard error of measurement. All experiment protocols were approved by the Animal Care and Use Committee of the University of Colorado at Boulder.

Healthy, non-obese rats, exposed to an acute stressor (tail shock) have elevated inflammatory protein concentrations in WAT [14]. Our lab has measured increases in IL- 1β (IL-1β), tumor necrosis factor-alpha (TNF-α), and interleukin-1 receptor antagonist (IL-1RA). Importantly only the IL-1β protein was affected in a depot selective fashion. More explicitly, stressor exposure increased IL-1β 5-fold in subcutaneous but not visceral WAT (Figure 2). We have therefore chosen to focus on IL-1β as a potential link between repeated stress and the development of visceral obesity. Within this framework, the data presented in Figure 2 lead to two important questions: 1) what is the functional significance of a regionally specific increase in subcutaneous IL-1β following acute stressor exposure and 2) how might repeated activation of this response play a role in the development of visceral obesity? Whereas the acute activation of this response likely serves beneficial functions, we hypothesize that repeated induction of this depot specific response may dampen the ability of non-visceral adipose tissue to absorb, retain lipids and/or expand via hyperplasia. Consequently visceral adipose deposition is increased, thus yielding potential mechanisms whereby stress-induced IL-1β signaling may affect body fat distribution. These hypotheses are further discussed in the final sections of this review.

### Potential adaptive functions of the acute IL-1β stress response in non-visceral adipose tissue

Elevations of inflammatory proteins in response to acute stressor exposure is part of the adaptive stress response and likely functions both locally (i.e. within tissues and organs) and systemically [109]. Cytokines such as IL-1β are best known for their role as immune modulators but they also affect local tissue functions such as adipose metabolism [100,110] suggesting that an acute, stress-induced rise in subcutaneous IL-1β content serves multiple functions within WAT. Given that this stress-induced IL-1β response occurs in the absence of a pathogen and in the WAT of healthy, non-obese rats, this cytokine may serve a non-traditional, metabolic function rather than acting as a traditional pro-inflammatory protein.

IL-1β is synthesized as a biologically inactive pro-protein following the activation and translocation of a transcription factor such as nuclear factor-kappa beta (NF-кB) [111,112]. Following activating signals that remain debated, pro-IL-1β is loaded onto a multi-protein platform called an inflammasome where it is cleaved by the IL-1β converting enzyme (caspase-1), converted into the mature form of IL-1β and released from the cell [112-114]. Because the cleavage of pro-IL-1β is thought to be followed by immediate release from its cellular source [115,116] and the ELISA kit (R&D Systems, Minneapolis, MN) used to quantify our preliminary data detects the mature form of the IL-1β protein [117], it is suggested that the measurable increase in WAT IL-1β reflects the release of mature IL-1β into the extracellular milieu of the tissue. In support of this theory, we have data demonstrating that the IL-1β measured in non-visceral WAT is not due to blood found in the tissue. Briefly, compared to non-perfused animals, rats exposed to tail shock and saline perfused (to remove blood from tissues) had equal increases in IL-1β content in their subcutaneous WAT (data not shown). Combined with evidence that the protein does not diffuse passively through the vascular endothelium [106,118] these data strongly suggest that IL-1β serves an autocrine and/or paracrine role within WAT [119]. Furthermore, since the primary functions of WAT are to store energy and act as an endocrine organ, stress-induced IL-1β signaling likely affects the metabolic and/or endocrine functions of WAT; however, because WAT also contains immune cells, mast cells, endothelial cells, preadipocytes and fibroblasts, IL-1β may also modulate a host of other functions. We therefore hypothesize that the stress-induced release of IL-1β within non-visceral WAT may serve metabolic and/or immunological functions that act in concert to fuel the high-energy demands of stress and promote host survival. The following are 4 ways in which stress-induced IL-1β could have these effects in non-visceral WAT.

#### Potentiates lipolysis in mature adipocytes

Multiple studies demonstrate that IL-1β has stimulating effect on lipolysis, or the liberation of free fatty acids and glycerol from mature adipocytes [120-123]. Stress induced free fatty acid release provides an oxidative fuel source for potentially active muscles and glycerol as a substrate for gluconeogenesis for the maintenance of blood glucose. IL-1β promotes lipolysis indirectly by reducing the production and/or activity of proteins that suppress lipolysis such as the lipid droplet-associated fat specific protein 27 (FSP27) [124] and lipoprotein lipase [120,125,126]. Reports have also shown that IL1β is able to induce changes in leptin secretion [127,128] which potentiates lipolysis through its inhibitory actions on insulin [129].

#### Potentiates stress-induced leptin release

We have preliminary data that acute stressor exposure induces leptin release both locally and systemically as marked by an increase in WAT and blood leptin concentrations (data not shown). Since leptin secreting adipocytes predominantly exist in the large, non-visceral, subcutaneous depots [130] and acute IL-1β signaling induces leptin secretion [127,128], stress-induced IL-1β in non-visceral WAT depots may potentiate leptin release. Leptin may subsequently act centrally to inhibit food intake, stimulate metabolic rate, and/or peripherally inhibit insulin action in adipocytes [129] - all of which would be appropriate and adaptive responses to an acute stressor. Moreover, leptin-suppressed insulin action in adipocytes would attenuate lipogenesis and/or potentiate lipolysis in non-visceral WAT demonstrating a potential mechanism for the regulation of body fat distribution.

#### Potentiates glucocorticoid activity

Evidence also suggests that, through its stimulating effect on the 11-beta hydroxysteroid dehydrogenase type 1 (11β-HSD1) enzyme which converts inactive glucocorticoids into their active form, IL-1β may indirectly increase local glucocorticoid activity [131,132]. This could consequentially serve beneficial metabolic and immunological functions in non-visceral WAT. For example, given that subcutaneous adipose tissue has a lower density of glucocorticoid receptors relative to visceral adipose tissue [11,133], subcutaneous-specific increases in glucocorticoid activity would help negate this difference. Metabolically this would be advantageous because glucocorticoids and insulin - both of which are released following acute stressor exposure relative to the severity of the stressor and the palatability of the diet available to the stressed subject [45] - are synergistic; glucocorticoids help insulin to promote lipid uptake and storage [134,135]. Increased depot specific/non-visceral, post-stress lipogenesis could then protect against visceral obesity by reinstating and maintaining the mass of the non-visceral depots. (Note: Because circulating levels of glucose and free fatty acids are tightly regulated, stress induced energy substrates must be removed from the blood either via utilization or uptake and re-storage. The term ‘post-stress lipogenesis’, therefore, refers to the process of removing stress-induced substrates from the blood for storage as triglycerides within WAT. Hence, following stress, the WAT depots with the highest lipogenic potential will serve as the primary re-deposition sites for unused substrates.)

Second, the presence of IL-1β in sub-dermal tissues such as subcutaneous WAT may immunologically “prime” the organism to effectively and efficiently combat a sub-dermal infection and/or injury [30,136]. In this case, potentiated glucocorticoid signaling in these depots would serve to boost the anti-inflammatory effects of glucocorticoids thus ensuring efficient regulation of stress-induced immune responses. Briefly, IL-1β acts as a pro-inflammatory protein by activating the transcription factor nuclear factor-kappa beta (NF-kβ) which initiates the synthesis of inflammatory proteins. In the case of a non-pathogenic stressor such as tail shock, a tightly regulated innate immune response would benefit the organism not only by conserving energy - an activated immune system can be metabolically demanding [137] - but by preserving the life and function of innate immune cells such as neutrophils and monocytes.

#### Promotes lipogenesis in resident macrophage cell membranes

IL-1β signaling may also play a critical role in the regulation of lipogenesis in the lipid bilayer of macrophage cell membranes. Im et al. (2011) demonstrated that macrophages from mice that don’t express sterol regulatory element binding protein-1a ((SREBP-1a) a nutrient sensing transcription factor) failed to activate lipogenesis and the release of IL-1β following a lipopolysaccharide challenge suggesting that IL-1β plays a central role in the link between lipid metabolism and the innate immune response [138].

**Figure 3:**
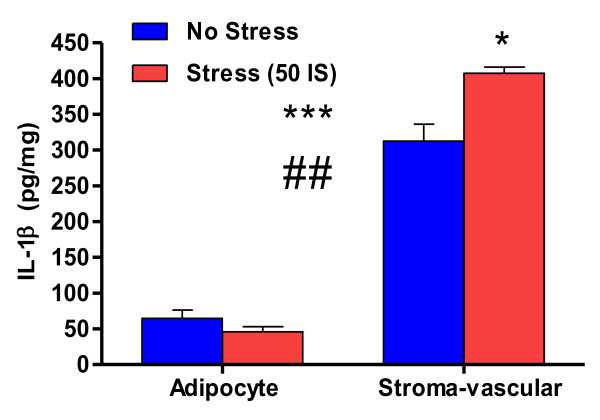
**IL-1β response to acute stressor exposure in adipocyte and stroma-vascular fractions of subcutaneous WAT.** Effect of acute stressor exposure on the IL-1β protein content of stromal-vascular and adipocyte fractions of subcutaneous (inguinal) WAT in healthy, non-obese rats. 21 week old male F344 rats were exposed to 50 inescapable 1.5 mA tail shocks (stress, n = 3) or remained in their home cages (no stress, n = 3). Rats were sacrificed immediately following stressor exposure. Approximately 0.3 g of subcutaneous WAT was harvested and digested via standard WAT collagenase digestion [139]. The stroma-vascular and adipocyte fractions were then processed via homogenization in a RIPA lysis buffer. IL-1β protein analysis (pg IL-1β per mg total protein) was done via ELISA (R&D Systems Minneapolis, MN). Effect of fraction on IL-1β concentration F(1,8) = 15.751, ***P < 0.0001. Effect of stress on IL-1β content: F(1,8) = 7.031, *P < 0.05. Fraction by stress interaction: F(3,6) = 15.697, ##P < 0.01. Values represent group means + standard error of measurement. All experiment protocols were approved by the Animal Care and Use Committee of the University of Colorado at Boulder.

These data also suggest that resident WAT macrophages are a cellular source of stress- induced IL-1β and that the SREBP-1a transcription factor may be involved in the sensing and transducing of the nutrient changes associated with acute stress in the local WAT environment. Moreover, Altintas et al. (2011) recently explored the distribution of mast cells and resident macrophages in subcutaneous and visceral fat of mice and found that resident macrophages were more prevalent than mast cells in lean WAT demonstrating that healthy, non-obese WAT does, in fact, contain resident macrophages [80]. Our lab also has data demonstrating that the highest concentration of basal and stress-induced IL- 1β lay within the stromal-vascular fraction of WAT (Figure 3). These data, however, do not reveal that resident macrophages per se are the cellular source of stress-induced IL- 1β. More work is necessary to determine the precise cellular sources of stress-induced IL-1β as other cells found in the stromal-vascular fraction of WAT likely contribute to the stress-induced IL-1β pool.

In summary, following acute stressor exposure, depot specific induction of IL-1β release may serve adaptive functions including potentiated lipolysis, leptin release and glucocorticoid activity. While a short-lived rise in subcutaneous WAT IL-1β is likely beneficial in nature, we propose the opposite holds true when repeatedly induced [105,109]. Hence the final section of this review presents evidence suggesting that repeated exposure to IL-1β in subcutaneous WAT may elicit maladaptive effects that could collectively contribute to the development of visceral obesity.

### Potential IL-1β related mechanisms whereby repeated stressor exposure induces a maladaptive shift in body fat distribution

Lipogenesis, or the process of removing energy substrates from the bloodstream and synthesizing them into a triglyceride molecule (i.e. three free fatty acids attached to a glycerol backbone), is the foundation upon which WAT stores and retains energy. The process of cleaving triglyceride molecules and liberating them as free fatty acids for energy utilization is termed lipolysis. The balance between these two processes, or lipolytic flux, determines the mean size of the mature adipocytes within the depot [76].

**Figure 4:**
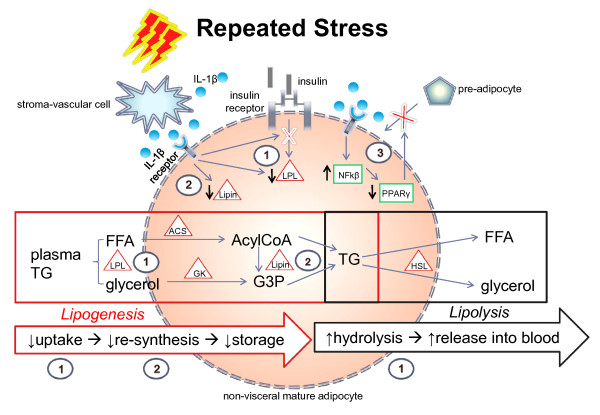
**Repeated stress-induced IL-1β may contribute to the development of visceral obesity through impairments in non-visceral WAT function.** Repeated exposure to acute stress may induce a maladaptive shift in the inflammatory milieu of subcutaneous adipose tissue marked by a repeated, regionally specific rise in IL-1β. In turn, this may lead to subcutaneous dysfunction or impairments in the depots’ ability to 1) uptake lipids, 2) resynthesize and/or retain lipids, and 3) expand via hyperplasia in the face of positive energy balance. These IL-1β-induced WAT dysfunctions may occur through the following mechanisms: 1) reduced lipoprotein lipase (LPL) activity - a decrease in LPL activity reduces lipid uptake which reduces triglyceride re-synthesis and storage and increases circulating lipid concentrations; 2) reduced Lipin-1 expression - decreased Lipin-1 expression negatively affects triglyceride re-synthesis and storage which further increases net circulating lipid concentrations; 3) reduced adipogeneic potential. IL-1β signaling activates the transcription factor NF-kβ which then reduces PPARγ activity. A reduction in PPARγ impairs adiopgenesis, or the differentiation of preadipocytes into mature, lipid storing, adipocytes. Collectively these effects may contribute toward the development of visceral obesity by a) reducing the size of non-visceral adipose depots relative to visceral adipose due to an imbalance in lipolytic flux (lipolysis > lipogenesis) and/or b) by shunting circulating lipids to non-visceral WAT for deposition due to the increased concentration of circulating lipids and reduced lipogenic/adipogenic potential of non-visceral WAT. Abbreviations: TG - triglyceride; FFA - free fatty acid; LPL - lipoprotein lipase; ACS - AcylCoA synthase; GK- glycerol kinase; NFkβ- nuclear factor kappa beta; PPARγ - peroxisome proliferator-activated receptor gamma.

The protective nature and function of non-visceral adipose tissue is associated with its ability to uptake circulating energy substrate, synthesize and/or retain triglyceride [26] and to expand via hyperplasia during prolonged positive energy balance [139]. Conversely, the pathogenic nature of visceral adipose is related to its ability to expand via hypertrophy and/or hyperplasia. Dysfunction of WAT therefore implies that its lipogenic and/or adipogenic abilities become maladaptive relative to its location. Interestingly, recent data demonstrate that the local milieu of inflammatory proteins modulates lipid uptake and release [100] suggesting that inflammatory proteins such as IL-1β may impact body fat distribution through the sum of their local effects on adipose metabolism. Evidence also demonstrates that viscerally obese subjects have subcutaneous adipose tissue that is marked by decreased expression of lipogenic and adipogenic proteins [26,140-143]. In spite of this evidence, no clear mechanisms have been proposed to date for the means whereby these impairments occur. While it is possible that repeated stressor exposure may drive visceral fat expansion by increasing the capability of visceral WAT to expand via hypertrophy and/or hyperplasia, we pose that repeated stress-induced IL-1β signaling negatively affects subcutaneous adipose tissue by dampening its lipogenic and/or adipogenic function. We offer evidence for the following hypothetical mechanisms, each of which, through their negative affect on subcutaneous WAT function, could contribute to the development of visceral obesity (Figure 4). Notably, repeated stressor exposure also induces variations in glucose-homeostasis [144], however, further discussion on this topic is beyond the scope of this review.

#### Impaired lipogenesis

The capacity of adipose tissue to absorb circulating lipids is regulated by the local expression and activity of LPL [145]. Because LPL activity is known to vary between adipose depots, it is thought to play a major role in regulating the distribution of fat deposition [29]. As Rebuffe-Scrive et al. (1988) and Stanhope et al. (2011) have described, the development of visceral obesity is marked by a decrease in LPL activity in subcutaneous adipose tissue [90]. Interestingly, separate studies demonstrate not only that IL-1β signaling directly decreases the activity of the LPL enzyme [120,122,146] but that repeated exposure to the protein also reduces insulin sensitivity [121,147,148]. These data are important because LPL activity is largely under the control of insulin demonstrating that IL-1β can directly and indirectly affect the activity of LPL [144,149]. IL-1β is also known to induce leptin secretion which further reduces insulin signaling in adipocytes. The decrease in lipoprotein lipase activity found in the subcutaneous adipose of viscerally obese subjects may be therefore be due to impaired insulin signaling induced by repeated IL-1β signaling in chronically stressed, subcutaneous adipose tissue. It is important to state, however, that IL-1β’s effects on insulin signaling and leptin secretion appear to be time-dependent; an acute rise in IL-1β has little to no effect on insulin sensitivity and potentiates leptin secretion whereas repeated or sustained exposure to the cytokine impairs insulin signaling and leptin secretion [121,127,146,147,150]. These data highlight the significance of short-term versus repeated or sustained IL-1β signaling and illustrate its potentially maladaptive effect on adipocyte function.

Mature adipocyte as well as lipid droplet size - adipocyte size is directly correlated to the size of its lipid droplets [151] - have also been shown to modulate local adipose metabolism. Ranjit et al. (2011) recently demonstrated that the lipolytic action of IL-1β is accompanied by a marked decrease in lipid droplet size and the expression of lipid droplet-associated fat specific protein 27 (FSP27 - a lipolytic suppressor) in mouse adipocytes [124]. On the other hand, Boivin et al. (2007) examined adipocytes from omental and subcutaneous WAT of 33 men ranging in BMI from 24.6 to 79.1 kg/m2 and found no differences in basal or isoproterenol induced lipolysis values between the depots across waist circumference tertiles [152]. The authors speculate, however, that this could be partly explained by the fact that differences in adipocyte size are important determinants of regional differences in adipose metabolism and their subject’s adipocytes were similar in size both between depots and across waist circumference [152]. Thus, through its potential effect on lipid droplet size, these data substantiate the hypothesis that IL-1β may play a role in the regulation of regionally-specific lipolytic responses to stress.

Evidence also suggests that IL-1β may dampen the ability of an adipocyte to resynthesize triglyceride molecules. Lu et al. (2008) examined the impact of IL-1β on the regulation of Lipin-1 expression and activity - an essential enzyme for triglyceride synthesis - and found that the expression of Lipin-1 was suppressed by IL-1β in cultured 3T3-L1 adipocytes and in mouse adipose tissue [123]. These data therefore suggest that repeated depot specific IL-1β signaling could contribute to a reduction in triglyceride synthesis thus reducing its buffering capacity while promoting the release of free fatty acids into the circulation.

Finally, in the case of sustained IL-1β and glucocorticoid signaling - the consequence of which is dampened insulin signaling - the acute benefit of site-specific post-stress lipogenesis becomes lost. In other words, if repeated stressor exposure reduces the buffering capacity of the non-visceral depots by impairing insulin signaling, LPL activity, and Lipin-1 expression it follows that a consequential increase in visceral and/or ectopic fat deposition would occur.

#### Impaired lipid retention

While acute mobilization of fatty acids from WAT is adaptive for energy mobilization during acute stress, if lipolysis is not equally opposed by post-stress lipogenesis, dissolution of the depot occurs. Lipid re-synthesis and retention in non-visceral WAT, therefore, is essential for the prevention visceral obesity. The ability of subcutaneous adipose tissue to retain lipids depends on the degree to which it is signaled to undergo lipolysis. As presented earlier, evidence suggests that IL-1β may both directly [83-85] and indirectly promote lipolysis. When unmatched by lipogenesis due to a decrease in LPL activity, insulin signaling, or Lipin-1 activity, atrophy of the subcutaneous depot occurs. These data therefore suggest that stress-induced IL-1β may lead to the development of visceral obesity by impeding the ability of subcutaneous adipose tissue to effectively uptake energy substrate and to re-synthesize and retain triglyceride molecules.

#### Impaired adipogenesis

Expansion of a WAT depot occurs through hypertrophy of mature adipocytes and/or through hyperplasia. The creation of new, mature adipocytes, or adipogenesis, involves the replication and differentiation of preadipocyte cells into mature, lipid storing adipoctyes and recent data demonstrate that the degree to which mature adipocytes can be formed depends upon the phenotype of the preadipocyte found within the depot [79,153]. Isakson et al. (2009) have further established that the phenotype of resident preadipocytes can be changed based on the composition of the inflammatory proteins found in the depot [142]. Moreover, whereas healthy, non-obese omental WAT contains preadipocytes with a reduced capacity for replication and differentiation [79], the preferred modality for healthy subcutaneous WAT expansion is through adipogenesis [82,94,154]. Because non-visceral depots constitute the bulk of total body fat mass, it therefore follows that a diminutive reduction in the adipogenic potential of subcutaneous WAT would negatively affect body fat distribution [82,93,94,139]. In support of our hypothesis that IL-1β could dampen the adipogenic potential of subcutaneous WAT, Lu et al. (2010) reported that the presence of as little as 5.0 pg/mL of IL-1β (equivalent to basal concentrations in visceral rat WAT) in the culture medium of 3T3-L1 preadipocytes inhibited adipogenesis [155]. Lu et al. (2010) further demonstrated that preadipocyte differentiation was also impaired upstream of IL-1β at the level of NF-кB due to its effect on the adipogenic transcription factor, PPARγ [155]. These data support other studies in which a rise in NF-κB activity leads to a reduction in PPARγ expression/activity and impairments in adipogenesis [156,157]. In this manner stress-induced IL-1β signaling could drive ectopic lipid deposition and visceral fat expansion by reducing the adipogenic potential of subcutaneous WAT. Contrary to this postulation, however, are data from Weise et al. (2008) suggesting that IL-1β stimulates the expression of tissue inhibitor of metalloproteinase (TIMP)-1, a protein thought to promote preadipocyte differentiation [158] and shown to be elevated in the serum of viscerally obese subjects [159]. Though Weise et al. (2008) demonstrated increased TIMP-1 secretion from mature 3T3-L1 adipocyte cells, concentrations of 0.5-20.0 ng/mL of IL-1β were required to induce this response [160] which may be outside of the physiological range for WAT. For example, as shown in Figure 2, stressed subcutaneous WAT concentrations rose to only 0.02 ng/mg total tissue. In addition, Weise et al. (2008) failed to demonstrate that IL-1β induced TIMP-1 synthesis leads to an increase in preadipocyte differentiation [160]. Consequently, the physiological relevance of their in vivo data in this study is unclear.

Finally, because glucocorticoid activation and signaling is known to stimulate early preadipocyte differentiation [161], an IL-1β induced rise in glucocorticoid activity seemingly contradicts the hypothesis that repeated exposure to IL-1β impairs the adiopogenic potential of adipose tissue. Interestingly, however, a transgenic study done by Masuzaki et al. (2001) demonstrated that depot specific increases in 11β-HSD1 activity did not, in fact, stimulate the differentiation of preadipocytes in either subcutaneous or visceral adipose tissue [12]. Instead, gains in adiposity instigated through transgenic over-expression of the enzyme were predominantly due to hypertrophy of the visceral adipocytes [12] suggesting that the effects of this enzyme are site dependent. While the authors speculated that exaggerated visceral fat deposition was due to enhanced glucocorticoid receptor expression, they did not provide a reason for the lack of hyperplasia found in the depots [12]. Although inflammatory proteins were not assessed in this study, our data along with the aforementioned studies [155,156] suggest that a rise in IL-1β activity and/or the activity of its primary transcription factor NF-кB may be involved in modulating the site specific effects of 11β-HSD1 and/or glucocorticoids on preadipocyte differentiation and adipocyte metabolism.

Despite conflicting evidence it is clear that sustained impairment of subcutaneous expansion or an increase in the capacity of visceral preadipocytes to expand or replicate in the face of positive energy balance could have momentous consequences on body fat distribution. As to which of these maladaptive events occurs first and where, though, remains unclear. Current data demonstrate that the inherent differences among the phenotypes of cells found within WAT depots contribute toward the interdepot variations seen in response to intrinsic and extrinsic stimuli such as IL-1β. Future research must therefore aim to understand the unique and adaptive functions of WAT and WAT proteins relative to time and depot location.

## Conclusions

In closing, stressors are an unavoidable fact of life for every organism. Our apparent inability to adaptively minimize and cope with stressors, coupled with a highly palatable/contemporary diet, has contributed to the development of a viscerally obese population [162]. While there is substantial evidence to support the idea that chronic stress is associated with disproportionate gains in visceral adiposity, the mechanisms whereby this selective deposition pattern occurs remain unclear. What is more, many of the studies that have investigated the potential effects of chronic stressor exposure on visceral obesity have failed to accurately assess body fat distribution, making an accurate characterization of its effects difficult to decipher.

Recent evidence suggests that local inflammatory proteins modulate adipocyte function and may therefore play a role in the regulation of body fat distribution. We present new data that acute stressor exposure increases the concentration of mature IL-1β within subcutaneous white adipose tissue to a significantly greater extent than in visceral white adipose tissue of healthy, non-obese rats. Acutely, the rise in this inflammatory protein likely serves beneficial functions such as increased lipolysis, increased leptin secretion and potentiated glucocorticoid signaling. However, if IL-1β signaling is sustained through repeated stressor exposure it could contribute to gains in visceral fat mass by reducing the ability of the subcutaneous adipose tissue to uptake, synthesize and retain triglyceride, and/or expand via hyperplasia in the face of positive energy balance. Further exploration of these novel hypotheses promises to expand our understanding of the adaptive functions of inflammatory proteins in healthy, non-obese adipose tissue and the mechanisms through which subcutaneous adipose tissue function may contribute to the regulation of body fat distribution and the development of visceral obesity. If local cross-talk between the innate and metabolic systems contributes to the development of visceral obesity, understanding the mechanisms whereby this occurs could lead to the development of therapeutic targets for the prevention of visceral obesity, the metabolic syndrome, and its associated diseases.

## Competing interests

The authors declare no competing interests.

## Authors’ contributions

KJS and MF were responsible for manuscript and figure preparation. KJS collected and analyzed the data. Both authors have read and approved the final version of this manuscript.

## References

[B1] Yusuf S Hawken S Ounpuu S Bautista L Franzosi MG Commerford P Lang CC Rumboldt Z Onen CL Lisheng L Obesity and the risk of myocardial infarction in 27,000 participants from 52 countries: a case–control study Lancet 2005 366 1640 1649 16271645 10.1016/S0140-6736(05)67663-5

[B2] Canoy D Boekholdt SM Wareham N Luben R Welch A Bingham S Buchan I Day N Khaw KT Body fat distribution and risk of coronary heart disease in men and women in the European Prospective Investigation Into Cancer and Nutrition in Norfolk cohort: a population-based prospective study Circulation 2007 116 2933 2943 18071080 10.1161/CIRCULATIONAHA.106.673756

[B3] Canoy D Wareham N Luben R Welch A Bingham S Day N Khaw KT Serum lipid concentration in relation to anthropometric indices of central and peripheral fat distribution in 20,021 British men and women: results from the EPIC-Norfolk population-based cohort study Atherosclerosis 2006 189 420 427 16442545 10.1016/j.atherosclerosis.2005.12.027

[B4] Bjorntorp P Abdominal fat distribution and disease: an overview of epidemiological data Ann Med 1992 24 15 18 1575956 10.3109/07853899209164140

[B5] Gleeson M Bishop NC Stensel DJ Lindley MR Mastana SS Nimmo MA The anti-inflammatory effects of exercise: mechanisms and implications for the prevention and treatment of disease Nat Rev Immunol 2011 11 607 615 21818123 10.1038/nri3041

[B6] Bjorntorp P Hormonal regulation of visceral adipose tissue Growth Horm IGF Res 1998 8 Suppl B 15 17 10990131 10.1016/s1096-6374(98)80020-8

[B7] Bjorntorp P The regulation of adipose tissue distribution in humans Int J Obes Relat Metab Disord 1996 20 291 302 8680455

[B8] Bjorntorp P Do stress reactions cause abdominal obesity and comorbidities? Obes Rev 2001 2 73 86 12119665 10.1046/j.1467-789x.2001.00027.x

[B9] Rosmond R Dallman MF Bjorntorp P Stress-related cortisol secretion in men: relationships with abdominal obesity and endocrine, metabolic and hemodynamic abnormalities J Clin Endocrinol Metab 1998 83 1853 1859 9626108 10.1210/jcem.83.6.4843

[B10] Kyrou I Chrousos GP Tsigos C Stress, visceral obesity, and metabolic complications Ann N Y Acad Sci 2006 1083 77 110 17148735 10.1196/annals.1367.008

[B11] Rebuffe-Scrive M Lundholm K Bjorntorp P Glucocorticoid hormone binding to human adipose tissue Eur J Clin Invest 1985 15 267 271 3935457 10.1111/j.1365-2362.1985.tb00182.x

[B12] Masuzaki H Paterson J Shinyama H Morton NM Mullins JJ Seckl JR Flier JS A transgenic model of visceral obesity and the metabolic syndrome Science 2001 294 2166 2170 11739957 10.1126/science.1066285

[B13] Mathis D Shoelson SE Immunometabolism: an emerging frontier Nat Rev Immunol 2011 11 81 21469396 10.1038/nri2922 PMC4784680

[B14] Speaker KJ SA Herrera J Cox S Strong P Greenwood B Fleshner M Investigation of complex stressor exposure on metabolic and inflammatory proteins in plasma and white adipose tissue Brain, Behavior and Immmunity 2011 25 S233 S234

[B15] Flegal KM Carroll MD Ogden CL Curtin LR Prevalence and trends in obesity among US adults, 1999–2008 JAMA 2010 303 235 241 20071471 10.1001/jama.2009.2014

[B16] Kloting N Fasshauer M Dietrich A Kovacs P Schon MR Kern M Stumvoll M Bluher M Insulin-sensitive obesity Am J Physiol Endocrinol Metab 2010 299 E506 E515 20570822 10.1152/ajpendo.00586.2009

[B17] Carey DG Jenkins AB Campbell LV Freund J Chisholm DJ Abdominal fat and insulin resistance in normal and overweight women: Direct measurements reveal a strong relationship in subjects at both low and high risk of NIDDM Diabetes 1996 45 633 638 8621015 10.2337/diab.45.5.633

[B18] Karelis AD St-Pierre DH Conus F Rabasa-Lhoret R Poehlman ET Metabolic and body composition factors in subgroups of obesity: what do we know? J Clin Endocrinol Metab 2004 89 2569 2575 15181025 10.1210/jc.2004-0165

[B19] Ferrannini E Natali A Bell P Cavallo-Perin P Lalic N Mingrone G Insulin resistance and hypersecretion in obesity. European Group for the Study of Insulin Resistance (EGIR) J Clin Invest 1997 100 1166 1173 9303923 10.1172/JCI119628 PMC508292

[B20] Bjorntorp P Body fat distribution, insulin resistance, and metabolic diseases Nutrition 1997 13 795 803 9290093 10.1016/s0899-9007(97)00191-3

[B21] Fujimoto WY Bergstrom RW Boyko EJ Chen KW Kahn SE Leonetti DL McNeely MJ Newell LL Shofer JB Tsunehara CH Wahl PW Preventing diabetes–applying pathophysiological and epidemiological evidence Br J Nutr 2000 84 Suppl 2 S173 S176 11242464 10.1079/096582197388635

[B22] Cnop M Landchild MJ Vidal J Havel PJ Knowles NG Carr DR Wang F Hull RL Boyko EJ Retzlaff BM The concurrent accumulation of intraabdominal and subcutaneous fat explains the association between insulin resistance and plasma leptin concentrations: distinct metabolic effects of two fat compartments Diabetes 2002 51 1005 1015 11916919 10.2337/diabetes.51.4.1005

[B23] Thorne A Lonnqvist F Apelman J Hellers G Arner P A pilot study of long- term effects of a novel obesity treatment: omentectomy in connection with adjustable gastric banding Int J Obes Relat Metab Disord 2002 26 193 199 11850750 10.1038/sj.ijo.0801871

[B24] Despres JP Lemieux I Abdominal obesity and metabolic syndrome Nature 2006 444 881 887 17167477 10.1038/nature05488

[B25] Kissebah AH Krakower GR Regional adiposity and morbidity Physiol Rev 1994 74 761 811 7938225 10.1152/physrev.1994.74.4.761

[B26] Manolopoulos KN Karpe F Frayn KN Gluteofemoral body fat as a determinant of metabolic health Int J Obes (Lond) 2010 34 949 959 20065965 10.1038/ijo.2009.286

[B27] Tanko LB Bagger YZ Alexandersen P Larsen PJ Christiansen C Peripheral adiposity exhibits an independent dominant antiatherogenic effect in elderly women Circulation 2003 107 1626 1631 12668497 10.1161/01.CIR.0000057974.74060.68

[B28] Bjorntorp P Thrifty genes and human obesity. Are we chasing ghosts? Lancet 2001 358 1006 1008 11583771 10.1016/S0140-6736(01)06110-4

[B29] Abate N Garg A Heterogeneity in adipose tissue metabolism: causes, implications and management of regional adiposity Prog Lipid Res 1995 34 53 70 7644553 10.1016/0163-7827(94)00006-8

[B30] Klein J Permana PA Owecki M Chaldakov GN Bohm M Hausman G Lapiere CM Atanassova P Sowinski J Fasshauer M What are subcutaneous adipocytes really good for? Exp Dermatol 2007 16 45 70 17181636 10.1111/j.1600-0625.2006.00519_1.x

[B31] Kopelman PG Obesity as a medical problem Nature 2000 404 635 643 10766250 10.1038/35007508

[B32] Stevens J Katz EG Huxley RR Associations between gender, age and waist circumference Eur J Clin Nutr 2010 64 6 15 19738633 10.1038/ejcn.2009.101 PMC5909719

[B33] Kuk JL Saunders TJ Davidson LE Ross R Age-related changes in total and regional fat distribution Ageing Res Rev 2009 8 339 348 19576300 10.1016/j.arr.2009.06.001

[B34] Wong SL Katzmarzyk P Nichaman MZ Church TS Blair SN Ross R Cardiorespiratory fitness is associated with lower abdominal fat independent of body mass index Med Sci Sports Exerc 2004 36 286 291 14767252 10.1249/01.MSS.0000113665.40775.35

[B35] Ross R Dagnone D Jones PJ Smith H Paddags A Hudson R Janssen I Reduction in obesity and related comorbid conditions after diet-induced weight loss or exercise-induced weight loss in men. A randomized, controlled trial Ann Intern Med 2000 133 92 103 10896648 10.7326/0003-4819-133-2-200007180-00008

[B36] George V Tremblay A Despres JP Leblanc C Bouchard C Effect of dietary fat content on total and regional adiposity in men and women Int J Obes 1990 14 1085 1094 2086500

[B37] Stanhope KL Havel PJ Fructose consumption: considerations for future research on its effects on adipose distribution, lipid metabolism, and insulin sensitivity in humans J Nutr 2009 139 1236S 1241S 19403712 10.3945/jn.109.106641 PMC3151025

[B38] Stanhope KL Role of Fructose-Containing Sugars in the Epidemics of Obesity and Metabolic Syndrome Annu Rev Med 2012 63 329 343 22034869 10.1146/annurev-med-042010-113026

[B39] Chiolero A Faeh D Paccaud F Cornuz J Consequences of smoking for body weight, body fat distribution, and insulin resistance Am J Clin Nutr 2008 87 801 809 18400700 10.1093/ajcn/87.4.801

[B40] Larsson B Svardsudd K Welin L Wilhelmsen L Bjorntorp P Tibblin G Abdominal adipose tissue distribution, obesity, and risk of cardiovascular disease and death: 13 year follow up of participants in the study of men born in 1913 Br Med J (Clin Res Ed) 1984 288 1401 1404 10.1136/bmj.288.6428.1401 PMC1441047 6426576

[B41] Rosmond R Aetiology of obesity: a striving after wind? Obes Rev 2004 5 177 181 15458392 10.1111/j.1467-789X.2004.00151.x

[B42] Rosmond R Bjorntorp P Psychosocial and socio-economic factors in women and their relationship to obesity and regional body fat distribution Int J Obes Relat Metab Disord 1999 23 138 145 10078847 10.1038/sj.ijo.0800782

[B43] Pilgaard L Lund P Rasmussen JG Fink T Zachar V Comparative analysis of highly defined proteases for the isolation of adipose tissue-derived stem cells Regen Med 2008 3 705 715 18729795 10.2217/17460751.3.5.705

[B44] Maier SF Watkins LR Role of the medial prefrontal cortex in coping and resilience Brain Res 2010 1355 52 60 20727864 10.1016/j.brainres.2010.08.039 PMC2967290

[B45] Dallman MF Pecoraro N Akana SF La Fleur SE Gomez F Houshyar H Bell ME Bhatnagar S Laugero KD Manalo S Chronic stress and obesity: a new view of "comfort food" Proc Natl Acad Sci U S A 2003 100 11696 11701 12975524 10.1073/pnas.1934666100 PMC208820

[B46] Gunn RC Smoking clinic failures and recent life stress Addict Behav 1983 8 83 87 6880930 10.1016/0306-4603(83)90062-x

[B47] Cooper ML Russell M Skinner JB Frone MR Mudar P Stress and alcohol use: moderating effects of gender, coping, and alcohol expectancies J Abnorm Psychol 1992 101 139 152 1537960 10.1037//0021-843x.101.1.139

[B48] Brady KT Sonne SC The role of stress in alcohol use, alcoholism treatment, and relapse Alcohol Res Health 1999 23 263 271 10890823 PMC6760383

[B49] van Praag HM Can stress cause depression? World J Biol Psychiatry 2005 6 Suppl 2 5 22 16166019 10.1080/15622970510030018

[B50] Bjorntorp PA Overweight is risking fate Best Practice & Research Clinical Endocrinology & Metabolism 1999 13 47 69 10.1053/beem.1999.0006 10932676

[B51] Rosmond R Contribution of stress to the development of the metabolic syndrome. In memory of Per Bjorntorp (1931–2003) Lakartidningen 2004 101 1371 1375 15146663

[B52] Smyth J Ockenfels MC Porter L Kirschbaum C Hellhammer DH Stone AA Stressors and mood measured on a momentary basis are associated with salivary cortisol secretion Psychoneuroendocrinology 1998 23 353 370 9695136 10.1016/s0306-4530(98)00008-0

[B53] Bjorntorp P Holm G Rosmond R Hypothalamic arousal, insulin resistance and Type 2 diabetes mellitus Diabet Med 1999 16 373 383 10342336 10.1046/j.1464-5491.1999.00067.x

[B54] Bjorntorp P Holm G Rosmond R Neuroendocrine disorders cause stress- related disease. "Civilization syndrome" is a growing health problem Lakartidningen 1999 96 893 896 10089734

[B55] Kuczmarski RJ Flegal KM Campbell SM Johnson CL Increasing prevalence of overweight among US adults. The National Health and Nutrition Examination Surveys, 1960 to 1991 JAMA 1994 272 205 211 8022039 10.1001/jama.272.3.205

[B56] Kalia M Assessing the economic impact of stress–the modern day hidden epidemic Metabolism 2002 51 49 53 12040542 10.1053/meta.2002.33193

[B57] Wing RR Marcus MD Epstein LH Jawad A A "family-based" approach to the treatment of obese type II diabetic patients J Consult Clin Psychol 1991 59 156 162 2002132 10.1037//0022-006x.59.1.156

[B58] Wajchenberg BL Subcutaneous and visceral adipose tissue: their relation to the metabolic syndrome Endocr Rev 2000 21 697 738 11133069 10.1210/edrv.21.6.0415

[B59] Dickerson SS Gruenewald TL Kemeny ME When the social self is threatened: shame, physiology, and health J Pers 2004 72 1191 1216 15509281 10.1111/j.1467-6494.2004.00295.x

[B60] Adam TC Epel ES Stress, eating and the reward system Physiol Behav 2007 91 449 458 17543357 10.1016/j.physbeh.2007.04.011

[B61] Drewnowski A Specter SE Poverty and obesity: the role of energy density and energy costs Am J Clin Nutr 2004 79 6 16 14684391 10.1093/ajcn/79.1.6

[B62] Coccurello R D'Amato FR Moles A Chronic social stress, hedonism and vulnerability to obesity: lessons from rodents Neurosci Biobehav Rev 2009 33 537 550 18585781 10.1016/j.neubiorev.2008.05.018

[B63] Tamashiro KL Nguyen MM Sakai RR Social stress: from rodents to primates Front Neuroendocrinol 2005 26 27 40 15862183 10.1016/j.yfrne.2005.03.001

[B64] Day HE Nebel S Sasse S Campeau S Inhibition of the central extended amygdala by loud noise and restraint stress Eur J Neurosci 2005 21 441 454 15673443 10.1111/j.1460-9568.2005.03865.x PMC2430886

[B65] Thompson RS Strong PV Fleshner M Physiological Consequences of Repeated Exposures to Conditioned Fear. Behavior Sciences 2012 2 2 57 78 10.3390/bs2020057 PMC4217585 25379216

[B66] Johnson JD Campisi J Sharkey CM Kennedy SL Nickerson M Greenwood BN Fleshner M Catecholamines mediate stress-induced increases in peripheral and central inflammatory cytokines Neuroscience 2005 135 1295 1307 16165282 10.1016/j.neuroscience.2005.06.090

[B67] Ricart-Jane D Cejudo-Martin P Peinado-Onsurbe J Lopez-Tejero MD Llobera M Changes in lipoprotein lipase modulate tissue energy supply during stress J Appl Physiol 2005 99 1343 1351 15947029 10.1152/japplphysiol.00971.2004

[B68] Solomon MB Jankord R Flak JN Herman JP Solomon MB Jankord R Flak JN Herman JP Chronic stress, energy balance and adiposity in female rats Physiol Behav 2010 102 84 90 20932852 10.1016/j.physbeh.2010.09.024 PMC3991931

[B69] Epel E Jimenez S Brownell K Stroud L Stoney C Niaura R Are stress eaters at risk for the metabolic syndrome? Ann N Y Acad Sci 2004 1032 208 210 15677412 10.1196/annals.1314.022

[B70] Tamashiro KL Hegeman MA Nguyen MM Melhorn SJ Ma LY Woods SC Sakai RR Dynamic body weight and body composition changes in response to subordination stress Physiol Behav 2007 91 440 448 17512562 10.1016/j.physbeh.2007.04.004 PMC1986729

[B71] Tamashiro KL Nguyen MM Ostrander MM Gardner SR Ma LY Woods SC Sakai RR Social stress and recovery: implications for body weight and body composition Am J Physiol Regul Integr Comp Physiol 2007 293 R1864 R1874 17855491 10.1152/ajpregu.00371.2007

[B72] Jayo JM Shively CA Kaplan JR Manuck SB Effects of exercise and stress on body fat distribution in male cynomolgus monkeys Int J Obes Relat Metab Disord 1993 17 597 604 8242129

[B73] Shively CA Clarkson TB Regional obesity and coronary artery atherosclerosis in females: a non-human primate model Acta Med Scand Suppl 1988 723 71 78 3164976 10.1111/j.0954-6820.1987.tb05930.x

[B74] Shively CA Clarkson TB Social status and coronary artery atherosclerosis in female monkeys Arterioscler Thromb 1994 14 721 726 8172850 10.1161/01.atv.14.5.721

[B75] Youngstrom TG Bartness TJ Catecholaminergic innervation of white adipose tissue in Siberian hamsters Am J Physiol 1995 268 R744 R751 7900918 10.1152/ajpregu.1995.268.3.R744

[B76] Rebuffe-Scrive M Neuroregulation of adipose tissue: molecular and hormonal mechanisms Int J Obes 1991 15 Suppl 2 83 86 1794942

[B77] Bartness TJ Bamshad M Innervation of mammalian white adipose tissue: implications for the regulation of total body fat Am J Physiol 1998 275 R1399 R1411 9791054 10.1152/ajpregu.1998.275.5.R1399

[B78] Toyoda M Matsubara Y Lin K Sugimachi K Furue M Characterization and comparison of adipose tissue-derived cells from human subcutaneous and omental adipose tissues Cell Biochem Funct 2009 27 440 447 19691084 10.1002/cbf.1591

[B79] Tchkonia T Tchoukalova YD Giorgadze N Pirtskhalava T Karagiannides I Forse RA Koo A Stevenson M Chinnappan D Cartwright A Abundance of two human preadipocyte subtypes with distinct capacities for replication, adipogenesis, and apoptosis varies among fat depots Am J Physiol Endocrinol Metab 2005 288 E267 E277 15383371 10.1152/ajpendo.00265.2004

[B80] Altintas MM Azad A Nayer B Contreras G Zaias J Faul C Reiser J Nayer A Mast cells, macrophages, and crown-like structures distinguish subcutaneous from visceral fat in mice J Lipid Res 2011 52 480 488 21148461 10.1194/jlr.M011338 PMC3035684

[B81] Tchkonia T Giorgadze N Pirtskhalava T Thomou T DePonte M Koo A Forse RA Chinnappan D Martin-Ruiz C von Zglinicki T Kirkland JL Fat depotspecific characteristics are retained in strains derived from single human preadipocytes Diabetes 2006 55 2571 2578 16936206 10.2337/db06-0540

[B82] Joe AW Yi L Even Y Vogl AW Rossi FM Depot-specific differences in adipogenic progenitor abundance and proliferative response to high-fat diet Stem Cells 2009 27 2563 2570 19658193 10.1002/stem.190

[B83] Roca-Rivada A Alonso J Al-Massadi O Castelao C Peinado JR Seoane LM Casanueva FF Pardo M Secretome analysis of rat adipose tissues shows location-specific roles for each depot type J Proteomics 2011 74 1068 1079 21439414 10.1016/j.jprot.2011.03.010

[B84] Bjorntorp P "Portal" adipose tissue as a generator of risk factors for cardiovascular disease and diabetes Arteriosclerosis 1990 10 493 496 2196039

[B85] Catalano KJ Stefanovski D Bergman RN Critical role of the mesenteric depot versus other intra-abdominal adipose depots in the development of insulin resistance in young rats Diabetes 2010 59 1416 1423 20299478 10.2337/db08-0675 PMC2874702

[B86] Frayn KN Visceral fat and insulin resistance–causative or correlative? Br J Nutr 2000 83 Suppl 1 S71 S77 10889795 10.1017/s0007114500000982

[B87] Khnychenko LK Sapronov NS Role stress on obesity and energy balance Usp Fiziol Nauk 2010 41 64 71 20865938

[B88] Araujo EP Torsoni MA Velloso LA Hypothalamic inflammation and obesity Vitam Horm 2010 82 129 143 20472136 10.1016/S0083-6729(10)82007-2

[B89] Balistreri CR Caruso C Candore G The role of adipose tissue and adipokines in obesity-related inflammatory diseases Mediators Inflamm 2010 2010 802078 20671929 10.1155/2010/802078 PMC2910551

[B90] Rebuffe-Scrive M Krotkiewski M Elfverson J Bjorntorp P Muscle and adipose tissue morphology and metabolism in Cushing's syndrome J Clin Endocrinol Metab 1988 67 1122 1128 3142910 10.1210/jcem-67-6-1122

[B91] Stanhope KL Griffen SC Bremer AA Vink RG Schaefer EJ Nakajima K Schwarz JM Beysen C Berglund L Keim NL Havel PJ Metabolic responses to prolonged consumption of glucose- and fructose-sweetened beverages are not associated with postprandial or 24-h glucose and insulin excursions Am J Clin Nutr 2011 94 112 119 21613559 10.3945/ajcn.110.002246 PMC3127512

[B92] Fried SK Russell CD Grauso NL Brolin RE Lipoprotein lipase regulation by insulin and glucocorticoid in subcutaneous and omental adipose tissues of obese women and men J Clin Invest 1993 92 2191 2198 8227334 10.1172/JCI116821 PMC288398

[B93] DiGirolamo M Fine JB Tagra K Rossmanith R Qualitative regional differences in adipose tissue growth and cellularity in male Wistar rats fed ad libitum Am J Physiol 1998 274 R1460 R1467 9612415 10.1152/ajpregu.1998.274.5.R1460

[B94] Einstein FH Atzmon G Yang XM Ma XH Rincon M Rudin E Muzumdar R Barzilai N Differential responses of visceral and subcutaneous fat depots to nutrients Diabetes 2005 54 672 678 15734842 10.2337/diabetes.54.3.672

[B95] Peinado JR Jimenez-Gomez Y Pulido MR Ortega-Bellido M Diaz-Lopez C Padillo FJ Lopez-Miranda J Vazquez-Martinez R Malagon MM The stromal-vascular fraction of adipose tissue contributes to major differences between subcutaneous and visceral fat depots Proteomics 2010 10 3356 3366 20706982 10.1002/pmic.201000350

[B96] Miranda M Chacon MR Gutierrez C Vilarrasa N Gomez JM Caubet E Megiam A Vendrell J LMNA mRNA expression is altered in human obesity and type 2 diabetes Obesity (Silver Spring) 2008 16 1742 1748 18497734 10.1038/oby.2008.276

[B97] Faust IM Johnson PR Stern JS Hirsch J Diet-induced adipocyte number increase in adult rats: a new model of obesity Am J Physiol 1978 235 E279 E286 696822 10.1152/ajpendo.1978.235.3.E279

[B98] Ntambi JM Young-Cheul K Adipocyte differentiation and gene expression J Nutr 2000 130 3122S 3126S 11110885 10.1093/jn/130.12.3122S

[B99] Caspar-Bauguil S Cousin B Bour S Castiella L Penicaud L Carpene C Adipose tissue lymphocytes: types and roles J Physiol Biochem 2009 65 423 436 20358356 10.1007/BF03185938

[B100] Permana PA Menge C Reaven PD Macrophage-secreted factors induce adipocyte inflammation and insulin resistance Biochem Biophys Res Commun 2006 341 507 514 16427608 10.1016/j.bbrc.2006.01.012

[B101] Suganami T Nishida J Ogawa Y A paracrine loop between adipocytes and macrophages aggravates inflammatory changes: role of free fatty acids andtumor necrosis factor alpha Arterioscler Thromb Vasc Biol 2005 25 2062 2068 16123319 10.1161/01.ATV.0000183883.72263.13

[B102] Besedovsky H del Rey A Sorkin E Dinarello CA Immunoregulatory feedback between interleukin-1 and glucocorticoid hormones Science 1986 233 652 654 3014662 10.1126/science.3014662

[B103] Kopp A Buechler C Neumeier M Weigert J Aslanidis C Scholmerich J Schaffler A Innate immunity and adipocyte function: ligand-specific activation of multiple Toll-like receptors modulates cytokine, adipokine, and chemokine secretion in adipocytes Obesity (Silver Spring) 2009 17 648 656 19148127 10.1038/oby.2008.607

[B104] Juge-Aubry CE Henrichot E Meier CA Adipose tissue: a regulator of inflammation Best Pract Res Clin Endocrinol Metab 2005 19 547 566 16311216 10.1016/j.beem.2005.07.009

[B105] Black PH The inflammatory consequences of psychologic stress: relationship to insulin resistance, obesity, atherosclerosis and diabetes mellitus, type II Med Hypotheses 2006 67 879 891 16781084 10.1016/j.mehy.2006.04.008

[B106] Steptoe A Hamer M Chida Y The effects of acute psychological stress on circulating inflammatory factors in humans: a review and meta-analysis Brain Behav Immun 2007 21 901 912 17475444 10.1016/j.bbi.2007.03.011

[B107] Segerstrom SC Miller GE Psychological stress and the human immune system: a meta-analytic study of 30 years of inquiry Psychol Bull 2004 130 601 630 15250815 10.1037/0033-2909.130.4.601 PMC1361287

[B108] Pedersen BK Hoffman-Goetz L Exercise and the immune system: regulation, integration, and adaptation Physiol Rev 2000 80 1055 1081 10893431 10.1152/physrev.2000.80.3.1055

[B109] Moraska A Campisi J Nguyen KT Maier SF Watkins LR Fleshner M Elevated IL-1beta contributes to antibody suppression produced by stress J Appl Physiol 2002 93 207 215 12070207 10.1152/japplphysiol.01151.2001

[B110] Coppack SW Pro-inflammatory cytokines and adipose tissue Proc Nutr Soc 2001 60 349 356 11681809 10.1079/pns2001110

[B111] Rubartelli A Cozzolino F Talio M Sitia R A novel secretory pathway for interleukin-1 beta, a protein lacking a signal sequence EMBO J 1990 9 1503 1510 2328723 10.1002/j.1460-2075.1990.tb08268.x PMC551842

[B112] Eder C Mechanisms of interleukin-1beta release Immunobiology 2009 214 543 553 19250700 10.1016/j.imbio.2008.11.007

[B113] Petrilli V Dostert C Muruve DA Tschopp J The inflammasome: a danger sensing complex triggering innate immunity Curr Opin Immunol 2007 19 615 622 17977705 10.1016/j.coi.2007.09.002

[B114] Pedra JH Cassel SL Sutterwala FS Sensing pathogens and danger signals by the inflammasome Curr Opin Immunol 2009 21 10 16 19223160 10.1016/j.coi.2009.01.006 PMC2701640

[B115] Singer II Scott S Chin J Bayne EK Limjuco G Weidner J Miller DK Chapman K Kostura MJ The interleukin-1 beta-converting enzyme (ICE) is localized on the external cell surface membranes and in the cytoplasmic ground substance of human monocytes by immuno-electron microscopy J Exp Med 1995 182 1447 1459 7595215 10.1084/jem.182.5.1447 PMC2192189

[B116] Watkins LR Hansen MK Nguyen KT Lee JE Maier SF Dynamic regulation of the proinflammatory cytokine, interleukin-1beta: molecular biology for non-molecular biologists Life Sci 1999 65 449 481 10462074 10.1016/s0024-3205(99)00095-8

[B117] Dinarello CA ELISA kits based on monoclonal antibodies do not measure total IL-1 beta synthesis J Immunol Methods 1992 148 255 259 1564330 10.1016/0022-1759(92)90179-w

[B118] Patterson SM Matthews KA Allen MT Owens JF Stress-induced hemoconcentration of blood cells and lipids in healthy women during acute psychological stress Health Psychol 1995 14 319 324 7556035 10.1037//0278-6133.14.4.319

[B119] Waki H Tontonoz P Endocrine functions of adipose tissue Annu Rev Pathol 2007 2 31 56 18039092 10.1146/annurev.pathol.2.010506.091859

[B120] Doerrler W Feingold KR Grunfeld C Cytokines induce catabolic effects in cultured adipocytes by multiple mechanisms Cytokine 1994 6 478 484 7827285 10.1016/1043-4666(94)90074-4

[B121] Lagathu C Yvan-Charvet L Bastard JP Maachi M Quignard-Boulange A Capeau J Caron M Long-term treatment with interleukin-1beta induces insulin resistance in murine and human adipocytes Diabetologia 2006 49 2162 2173 16865359 10.1007/s00125-006-0335-z

[B122] Hardardottir I Doerrler W Feingold KR Grunfeld C Cytokines stimulate lipolysis and decrease lipoprotein lipase activity in cultured fat cells by a prostaglandin independent mechanism Biochem Biophys Res Commun 1992 186 237 243 1632769 10.1016/s0006-291x(05)80798-3

[B123] Lu B Lu Y Moser AH Shigenaga JK Grunfeld C Feingold KR LPS and proinflammatory cytokines decrease lipin-1 in mouse adipose tissue and 3T3–L1 adipocytes Am J Physiol Endocrinol Metab 2008 295 E1502 E1509 18940942 10.1152/ajpendo.90323.2008 PMC2603550

[B124] Ranjit S Boutet E Gandhi P Prot M Tamori Y Chawla A Greenberg AS Puri V Czech MP Regulation of fat specific protein 27 by isoproterenol and TNF- alpha to control lipolysis in murine adipocytes J Lipid Res 2011 52 221 236 21097823 10.1194/jlr.M008771 PMC3023542

[B125] Price SR Mizel SB Pekala PH Regulation of lipoprotein lipase synthesis and 3T3-L1 adipocyte metabolism by recombinant interleukin 1 Biochim BiophysActa 1986 889 374 381 10.1016/0167-4889(86)90201-6 3491626

[B126] Feingold KR Doerrler W Dinarello CA Fiers W Grunfeld C Stimulation of lipolysis in cultured fat cells by tumor necrosis factor, interleukin-1, and the interferons is blocked by inhibition of prostaglandin synthesis Endocrinology 1992 130 10 16 1370149 10.1210/endo.130.1.1370149

[B127] Bruun JM Pedersen SB Kristensen K Richelsen B Effects of pro-inflammatory cytokines and chemokines on leptin production in human adipose tissue in vitro Mol Cell Endocrinol 2002 190 91 99 11997182 10.1016/s0303-7207(02)00007-2

[B128] Gonzalez RR Leavis P Leptin upregulates beta3-integrin expression and interleukin-1beta, upregulates leptin and leptin receptor expression in human endometrial epithelial cell cultures Endocrine 2001 16 21 28 11822823 10.1385/ENDO:16:1:21

[B129] Muller G Ertl J Gerl M Preibisch G Leptin impairs metabolic actions of insulin in isolated rat adipocytes J Biol Chem 1997 272 10585 10593 9099705 10.1074/jbc.272.16.10585

[B130] Trayhurn P Hoggard N Mercer JG Rayner DV Leptin: fundamental aspects Int J Obes Relat Metab Disord 1999 23 Suppl 1 22 28 10193858 10.1038/sj.ijo.0800791

[B131] Friedberg M Zoumakis E Hiroi N Bader T Chrousos GP Hochberg Z Modulation of 11 beta-hydroxysteroid dehydrogenase type 1 in mature human subcutaneous adipocytes by hypothalamic messengers J Clin Endocrinol Metab 2003 88 385 393 12519881 10.1210/jc.2002-020510

[B132] Tomlinson JW Moore J Cooper MS Bujalska I Shahmanesh M Burt C Strain A Hewison M Stewart PM Regulation of expression of 11beta-hydroxysteroid dehydrogenase type 1 in adipose tissue: tissue-specific induction by cytokines Endocrinology 2001 142 1982 1989 11316764 10.1210/endo.142.5.8168

[B133] Pedersen SB Jonler M Richelsen B Characterization of regional and gender differences in glucocorticoid receptors and lipoprotein lipase activity in human adipose tissue J Clin Endocrinol Metab 1994 78 1354 1359 8200937 10.1210/jcem.78.6.8200937

[B134] Ottosson M Lonnroth P Bjorntorp P Eden S Effects of cortisol and growthhormone on lipolysis in human adipose tissue J Clin Endocrinol Metab 2000 85 799 803 10690893 10.1210/jcem.85.2.6358

[B135] Warne JP Akana SF Ginsberg AB Horneman HF Pecoraro NC Dallman MF Disengaging insulin from corticosterone: roles of each on energy intake and disposition Am J Physiol Regul Integr Comp Physiol 2009 296 R1366 R1375 19279289 10.1152/ajpregu.91016.2008 PMC2689821

[B136] Fernandez-Real JM Ricart W Insulin resistance and inflammation in an evolutionary perspective: the contribution of cytokine genotype/phenotype to thriftiness Diabetologia 1999 42 1367 1374 10550422 10.1007/s001250051451

[B137] Muehlenbein MP Hirschtick JL Bonner JZ Swartz AM Toward quantifying the usage costs of human immunity: Altered metabolic rates and hormone levels during acute immune activation in men Am J Hum Biol 2010 22 546 556 20309883 10.1002/ajhb.21045

[B138] Im SS Yousef L Blaschitz C Liu JZ Edwards RA Young SG Raffatellu M Osborne TF Linking lipid metabolism to the innate immune response in macrophages through sterol regulatory element binding protein-1a Cell metabolism 2011 13 540 549 21531336 10.1016/j.cmet.2011.04.001 PMC3090630

[B139] Mathieu P Lemieux I Despres JP Obesity, inflammation, and cardiovascular risk Clin Pharmacol Ther 2010 87 407 416 20200516 10.1038/clpt.2009.311

[B140] Jensen MD Role of body fat distribution and the metabolic complications of obesity J Clin Endocrinol Metab 2008 93 S57 S63 18987271 10.1210/jc.2008-1585 PMC2585758

[B141] Johnson JA Fried SK Pi-Sunyer FX Albu JB Impaired insulin action in subcutaneous adipocytes from women with visceral obesity Am J Physiol Endocrinol Metab 2001 280 E4049 10.1152/ajpendo.2001.280.1.E40 11120657

[B142] Isakson P Hammarstedt A Gustafson B Smith U Impaired preadipocyte differentiation in human abdominal obesity: role of Wnt, tumor necrosisfactor-alpha, and inflammation Diabetes 2009 58 1550 1557 19351711 10.2337/db08-1770 PMC2699851

[B143] Bell ME Bhargava A Soriano L Laugero K Akana SF Dallman MF Sucrose intake and corticosterone interact with cold to modulate ingestive behaviour, energy balance, autonomic outflow and neuroendocrine responses during chronic stress J Neuroendocrinol 2002 14 330 342 11963830 10.1046/j.1365-2826.2002.00784.x

[B144] Eckel RH Lipoprotein lipase. A multifunctional enzyme relevant to common metabolic diseases N Engl J Med 1989 320 1060 1068 2648155 10.1056/NEJM198904203201607

[B145] Beutler BA Cerami A Recombinant interleukin 1 suppresses lipoprotein lipase activity in 3 T3-L1 cells J Immunol 1985 135 3969 3971 2999235

[B146] Hube F Hauner H The role of TNF-alpha in human adipose tissue: prevention of weight gain at the expense of insulin resistance? Horm Metab Res 1999 31 626 631 10668912 10.1055/s-2007-978810

[B147] Jager J Gremeaux T Cormont M Le Marchand-Brustel Y Tanti JF Interleukin-1beta-induced insulin resistance in adipocytes through downregulation of insulin receptor substrate-1 expression Endocrinology 2007 148 241 251 17038556 10.1210/en.2006-0692 PMC1971114

[B148] Sadur CN Eckel RH Insulin stimulation of adipose tissue lipoprotein lipase. Use of the euglycemic clamp technique J Clin Invest 1982 69 1119 1125 7040473 10.1172/JCI110547 PMC370176

[B149] Matsuki T Horai R Sudo K Iwakura Y IL-1 plays an important role in lipid metabolism by regulating insulin levels under physiological conditions J Exp Med 2003 198 877 888 12975454 10.1084/jem.20030299 PMC2194201

[B150] Suzuki M Shinohara Y Ohsaki Y Fujimoto T Lipid droplets: size matters J Electron Microsc (Tokyo) 2011 60 Suppl 1 S101 S116 21844583 10.1093/jmicro/dfr016

[B151] Boivin A Brochu G Marceau S Marceau P Hould FS Tchernof A Regional differences in adipose tissue metabolism in obese men Metabolism 2007 56 533 540 17379013 10.1016/j.metabol.2006.11.015

[B152] Tchkonia T Lenburg M Thomou T Giorgadze N Frampton G Pirtskhalava T Cartwright A Cartwright M Flanagan J Karagiannides I Identification ofdepot-specific human fat cell progenitors through distinct expression profilesand developmental gene patterns Am J Physiol Endocrinol Metab 2007 292 E298 E307 16985259 10.1152/ajpendo.00202.2006

[B153] DiGirolamo M Fine JB Tagra K Rossmanith R Qualitative regional differences in adipose tissue growth and cellularity in male Wistar rats fed ad libitum Am J Physiol Regul Integr Comp Physiol 1998 274 R1460 R1467 10.1152/ajpregu.1998.274.5.R1460 9612415

[B154] Lu C Kumar PA Fan Y Sperling MA Menon RK A novel effect of growth hormone on macrophage modulates macrophage-dependent adipocyte differentiation Endocrinology 2010 151 2189 2199 20185763 10.1210/en.2009-1194 PMC2869256

[B155] Nunn AV Bell J Barter P The integration of lipid-sensing and anti-inflammatory effects: how the PPARs play a role in metabolic balance Nucl Recept 2007 5 1 17531095 10.1186/1478-1336-5-1 PMC1899481

[B156] Suzawa M Takada I Yanagisawa J Ohtake F Ogawa S Yamauchi T Kadowaki T Takeuchi Y Shibuya H Gotoh Y Cytokines suppress adipogenesis and PPAR-gamma function through the TAK1/TAB1/NIK cascade Nat Cell Biol 2003 5 224 230 12598905 10.1038/ncb942

[B157] Alexander CM Selvarajan S Mudgett J Werb Z Stromelysin-1 regulates adipogenesis during mammary gland involution J Cell Biol 2001 152 693 703 11266461 10.1083/jcb.152.4.693 PMC2195781

[B158] Kralisch S Bluher M Tonjes A Lossner U Paschke R Stumvoll M Fasshauer M Tissue inhibitor of metalloproteinase-1 predicts adiposity in humans Eur J Endocrinol 2007 156 257 261 17287416 10.1530/eje.1.02328

[B159] Weise S Kralisch S Sommer G Lossner U Bluher M Stumvoll M Fasshauer M Tissue inhibitor of metalloproteinase-1 mRNA production and protein secretion are induced by interleukin-1 beta in 3 T3-L1 adipocytes J Endocrinol 2008 198 169 174 18577571 10.1677/JOE-07-0631

[B160] Student AK Hsu RY Lane MD Induction of fatty acid synthetase synthesis in differentiating 3 T3-L1 preadipocytes J Biol Chem 1980 255 4745 4750 7372608

[B161] Dallman MF Pecoraro NC la Fleur SE Chronic stress and comfort foods: self- medication and abdominal obesity Brain Behav Immun 2005 19 275 280 15944067 10.1016/j.bbi.2004.11.004

[B162] Hochberg I Hochberg Z Expanding the definition of hypothalamic obesity Obes Rev 2010 11 709 721 20233310 10.1111/j.1467-789X.2010.00727.x

